# Characterization of Tensile Mechanical Behavior of MSCs/PLCL Hybrid Layered Sheet

**DOI:** 10.3390/jfb7020014

**Published:** 2016-06-03

**Authors:** Azizah Intan Pangesty, Takaaki Arahira, Mitsugu Todo

**Affiliations:** 1 Interdisciplinary Graduate School of Engineering Sciences, Kyushu University, Fukuoka 816-8580, Japan; azizah_intan@rocketmail.com; 2 Fukuoka Dental Collage, Fukuoka 814-0193, Japan; arahira@college.fdcnet.ac.jp; 3 Research Institute for Applied Mechanics, Kyushu University, Fukuoka 816-8580, Japan

**Keywords:** cell sheet technology, scaffold tissue engineering, PLCL sheet, MSCs sheet, tensile mechanical properties, tissue engineered patch

## Abstract

A layered construct was developed by combining a porous polymer sheet and a cell sheet as a tissue engineered vascular patch. The primary objective of this study is to investigate the influence of mesenchymal stem cells (MSCs) sheet on the tensile mechanical properties of porous poly-(l-lactide-co-ε-caprolactone) (PLCL) sheet. The porous PLCL sheet was fabricated by the solid-liquid phase separation method and the following freeze-drying method. The MSCs sheet, prepared by the temperature-responsive dish, was then layered on the top of the PLCL sheet and cultured for 2 weeks. During the *in vitro* study, cellular properties such as cell infiltration, spreading and proliferation were evaluated. Tensile test of the layered construct was performed periodically to characterize the tensile mechanical behavior. The tensile properties were then correlated with the cellular properties to understand the effect of MSCs sheet on the variation of the mechanical behavior during the *in vitro* study. It was found that MSCs from the cell sheet were able to migrate into the PLCL sheet and actively proliferated into the porous structure then formed a new layer of MSCs on the opposite surface of the PLCL sheet. Mechanical evaluation revealed that the PLCL sheet with MSCs showed enhancement of tensile strength and strain energy density at the first week of culture which is characterized as the effect of MSCs proliferation and its infiltration into the porous structure of the PLCL sheet. New technique was presented to develop tissue engineered patch by combining MSCs sheet and porous PLCL sheet, and it is expected that the layered patch may prolong biomechanical stability when implanted *in vivo*.

## 1. Introduction

Many patients with congenital heart disease are often required to have surgical intervention to close the defect with patch graft [1,2]. Unfortunately, the growth potential of currently available patches is low [3,4,5] with several problems. For example, prosthetic and bovine pericardium patches are potentially calcified due to tissue incompatibility [6,7,8]. Autologous pericardium patches are known for their superior biocompatibility, lower risk of contamination, and low cost [9]. However, the use of fresh autologous pericardium is limited due to handling difficulty and its potential for shrinking or stretching after transplantation [6,10].

Currently, tissue engineering approaches have a great potential for providing vascular patch with capacity to grow and regenerate [2]. In attempt to developing tissue engineered patch, biodegradable polymers such as poly glycolic acid (PGA) [11,12,13] and poly caprolactone (PCL) [13,14,15,16] are often used to fabricate the patch scaffolds. PLCL draws special interest mainly because its mechanical properties are very close to those of native cardiovascular tissue [17,18,19,20]. Vascular tissue is constructed by multilayer of cells which align circumferentially in a complex anatomy [21]. Ideally, the seeded cells are able to grow and regenerate mature tissue layers resembled to native vascular structure along with the degradation of patch scaffold [22]. However, regenerating a complex structure-like vascular through patch tissue engineering have yet successfully achieved in clinical application.

Such mature layer of cells can be prepared *in vitro* using cell sheet technology [23]. Facilitated by the temperature responsive culture dishes that are created by the covalent grafting of the temperature responsive polymer poly(*n*-isopropylacrylamide) (PIPAAm), confluent cells can be harvested as an intact layer simply by reducing its temperature below 32 °C [24,25]. As cells remain confluent, the critical components of the extracellular matrix such as growth factor protein, adhesion protein, and cell-to-cell junction protein remain intact. Previously, cell sheets constructed from MSCs have been reported to repair partial tissue defect such as scarred myocardium [26] and has capacity to differentiate not only into osteochondral cell but also into vascular endothelial cell [27]. Through this method, tissue engineered patch composed of multilayered cells can be potentially constructed. However, the construction of a cell-dense tissue based on cell sheets has remained a difficulty due to hypoxia, waste accumulation and the lack of nutrients, all to be expected from cell tissue with no circulatory support [28,29,30].

In this study, we investigated a new approach to develop a tissue engineered patch by combining the scaffold tissue engineering and the cell sheet technology that may offer a mutual benefit between the two methods. We attempted to develop a hybrid construct by layering a MSCs sheet on a porous PLCL sheet for the tissue engineered patch. During 2 weeks of *in vitro* course, cell spreading behavior and cellular infiltration on scaffold were evaluated by scanning electron microscopy and histological staining, respectively. We also assessed cell proliferation on scaffold. Furthermore, tensile tests of the PLCL sheet with and without MSCs sheet were performed periodically. Finally, the change of tensile properties was correlated with the cellular properties to understand the mechanism of variational behavior of the tensile mechanical properties.

## 2. Results

### 2.1. Morphology and Microstructure of PLCL Sheet and MSCs Sheet

**Figure 1:**
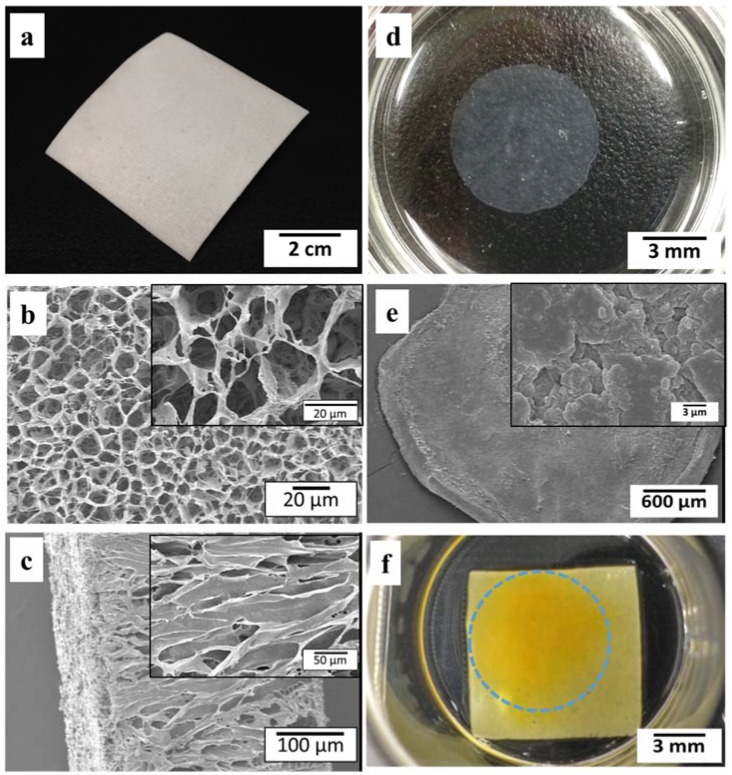
(**a**) Gross appearance of PLCL sheet; Porous microstructure of PLCL sheet at (**b**) surface area and (**c**) cross section; (**d**) MSCs sheet after detachment; (**e**) SEM images of MSNs sheet; (**f**) layered construct of PLCL sheet and MSCs sheet, yellow indicated cell sheet. Inset image show higher magnification.

In this experiment, the fabricated tubular graft from PLCL was cut into a sheet (Figure 1a). The thickness of PLCL sheet was around 0.5 mm. SEM images (Figure 1b) showed that the PLCL sheet consists of porous structure with an average pore size of 21 µm ± 4.5 µm. The cross sectional image (Figure 1c) showed elongated pores connected to each other, indicated that PLCL sheet was very interconnecting.

Cell sheet was prepared by seeding MSCs on the temperature-responsive dish. After 4 days of culture, the MSCs sheet was spontaneously detached at room temperature within 20 min and shrank to approximately 25% of its original diameter (Figure 1d). SEM observation of the MSCs sheet (Figure 1e) revealed an intact cell sheet with abundance of extracellular matrix. It was difficult to observe each cells distinctly due to high cell density. The bumpy surface morphology was created as a result of cells sheet contraction after detachment.

For fabrication of hybrid construct, one layer of MSCs sheet was layered on PLCL sheet (10 mm × 10 mm in size) using pipet. Figure 1f showed gross appearance of the hybrid layered construct with MSCs sheet remained attached (as indicated in yellow) at 7 days of culture.

### 2.2. Layered Construct of PLCL Sheet and MSCs Sheet

#### 2.2.1. Cell Spreading and Infiltration

**Figure 2:**
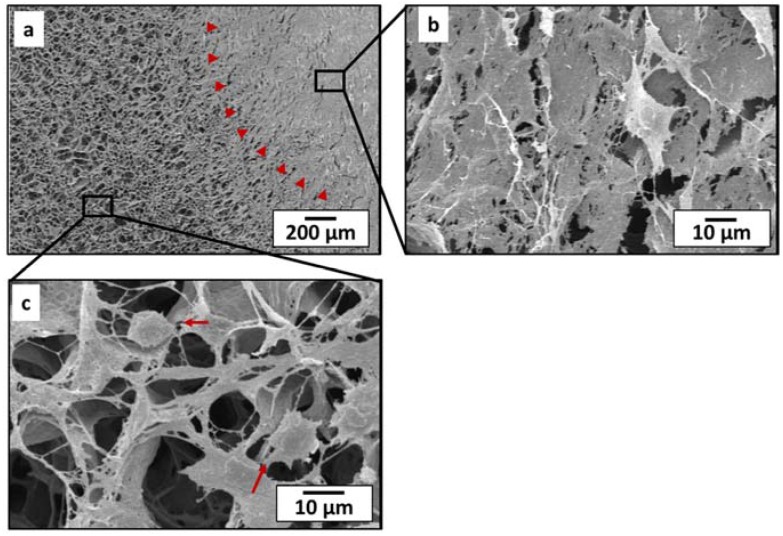
SEM images of cell spreading behavior on PLCL sheet at 14 days of culture. (**a**) Cell sheet attached (head arrows indicates MSCs sheet) and (**b**) covered the porous structure of PLCL sheet; (**c**) It also proliferates horizontally by creating a connecting network (arrows indicate cells).

To observe the behavior of cell sheet spreading on the PLCL sheet, SEM imaging was performed. SEM images of surface region at 14 days of culture were shown in Figure 2. The cell sheet (indicated by the red head arrows) covered half surface of the PLCL sheet (Figure 2a), making the pore structure of the PLCL sheet completely unobservable (Figure 2b). The cell sheet was able to proliferate actively across the entire surface of the polymer sheet by creating a network structure of extracellular matrix (Figure 2c).

**Figure 3:**
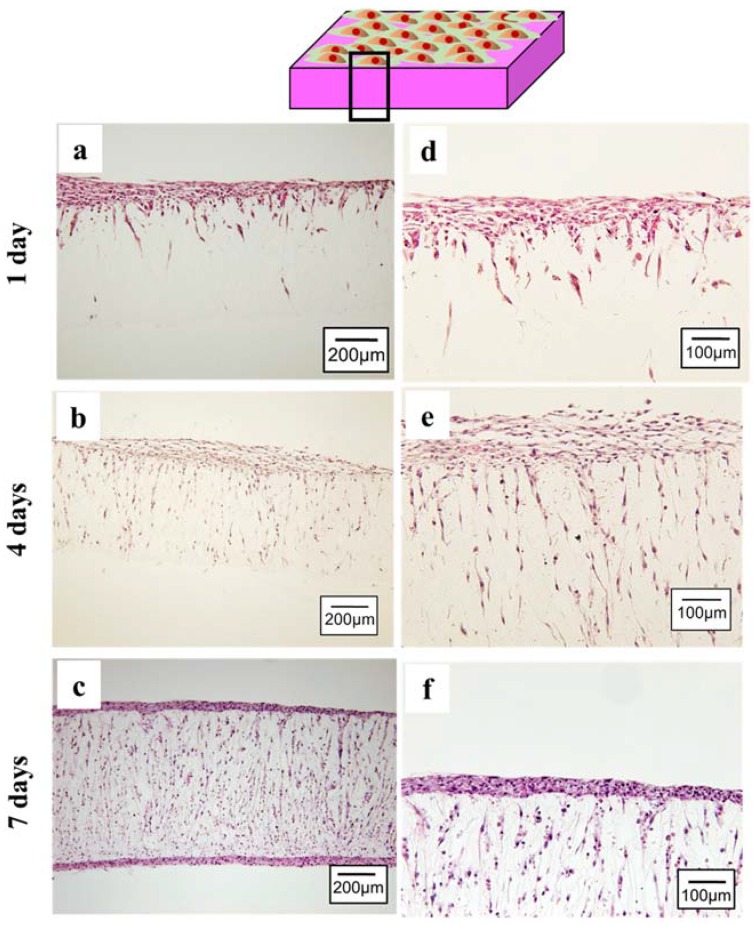
Histological observation of PLCL sheet with MSCs sheet at (**a**,**d**) 1 day; (**b**,**e**) 4 days and (**c**,**f**) 7 days of culture. Right panel shows higher magnification.

An ideal scaffold can promote cell infiltration and thus encourage the regeneration of new tissue. The infiltration of cells from the MSCs sheet was examined by histological staining during the *in vitro* course. The cross sectional image at 1 day culture showed that the MSCs sheet well attached on the PLCL sheets (Figure 3a). It is clearly seen that cells from the MSCs sheet migrate inside the PLCL sheet through connecting pores (Figure 3d). After 4 days of culture, the cells had continued to proliferate deeper through the connecting pores as shown in Figure 3b,e. After 7 days of culture, a new layer-like cell sheet had formed on the bottom side of the polymer sheet, resulting in a unique, sandwich-like structure (Figure 3c,f).

#### 2.2.2. Cell Proliferation

**Figure 4:**
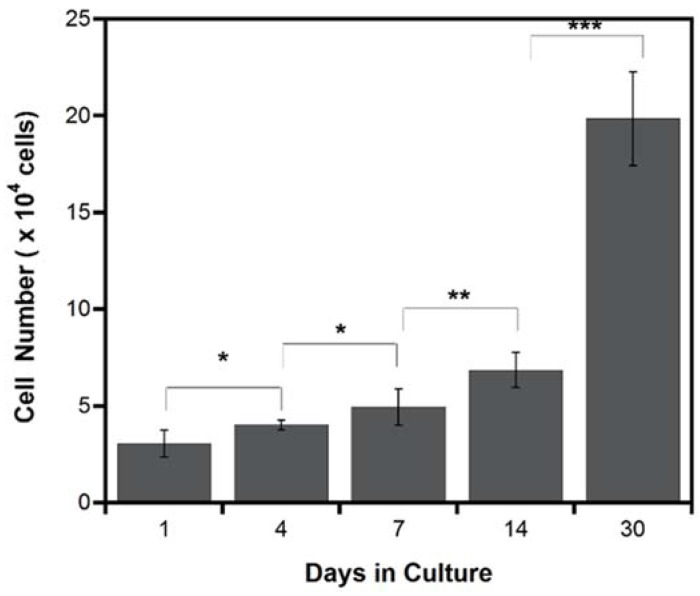
The number of viable cells in hybrid graft determined by cell counting kit during 30 days of culture. Each data represented mean ± SD, *n* = 4, * *p* < 0.05, ** *p* < 0.001, *** *p* < 0.0001.

Cell proliferation is a critical part for the complete cellularization of patch graft and a good indication for tissue regeneration. Cell proliferation on the PLCL sheet was examined quantitatively at 1, 4, 7, 14 and 30 days of culture using the cell counting kit which contains water soluble tetrazolium salt, generating yellow color from only viable cells. As shown in Figure 4, MSCs actively proliferated in significant amount from day 1 to day 14. At day 30, the number of live cells increased more than 2 folds from day 14. This experiment therefore gives strong evidence that the PLCL sheets are compatible for cell growing.

#### 2.2.3. Tensile Mechanical Evaluation

**Figure 5:**
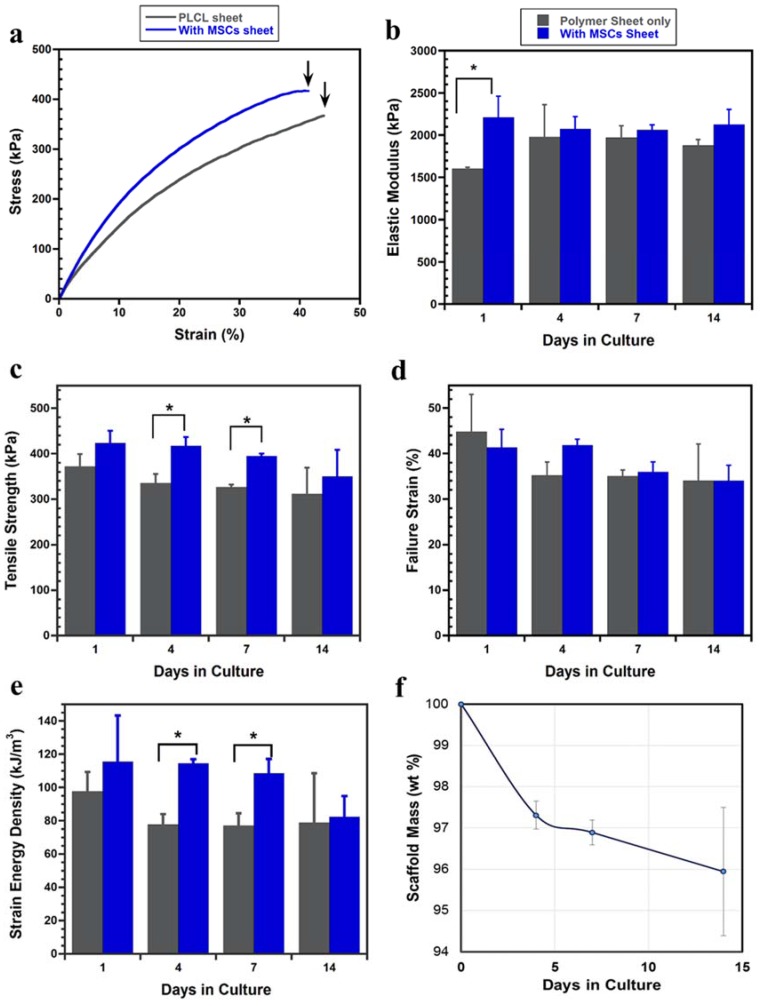
(**a**) Typical stress-strain curve at 1 day culture. Black arrow indicate point of breaking; (**b**) Comparison of average elastic modulus; (**c**) Comparison of averaged ultimate tensile strength; (**d**) Averaged of failure strain; (**e**) Averaged of strain energy density; (**f**) Data represent as mean ± SD, *n* = 3, * *p* < 0.05.

To evaluate the mechanical properties of both the PLCL sheet with and without MSCs, tensile tests were performed. The PLCL sheet with MSCs exhibited steeper stress-strain curves compared to the PLCL sheet only as shown in Figure 5a. In Figure 5b, the PLCL sheet with the cell sheet showed an increase of elastic modulus significantly at day 1 culture. However, the elastic moduli of the PLCL sheet with MSCs from 4 days to 14 days were insignificantly different from that of the PLCL sheet only. Figure 5c showed the averaged ultimate tensile strength during culture. At 1 day culture, the effect of the MSCs sheet on the tensile strength of the PLCL sheet was less apparent, however, the PLCL sheet with MSCs showed improvement of the tensile strength in significant amount at 4 and 7 days of culture. The differences of failure strain between the PLCL sheets with and without MSCs were not obvious during the cell culture period (Figure 5d). It was also found that the strain energy density of the PLCL sheet with MSCs showed a significant enhancement compared with the PLCL sheet at 4 and 7 days culture (Figure 5e). In general, it can be recognized that there was tendency of decreasing the mechanical properties at 14 days of culture. The scaffold mass evaluation revealed that the PLCL sheet lost its weight around 4% after 14 days incubation, as shown in Figure 5f.

**Figure 6:**
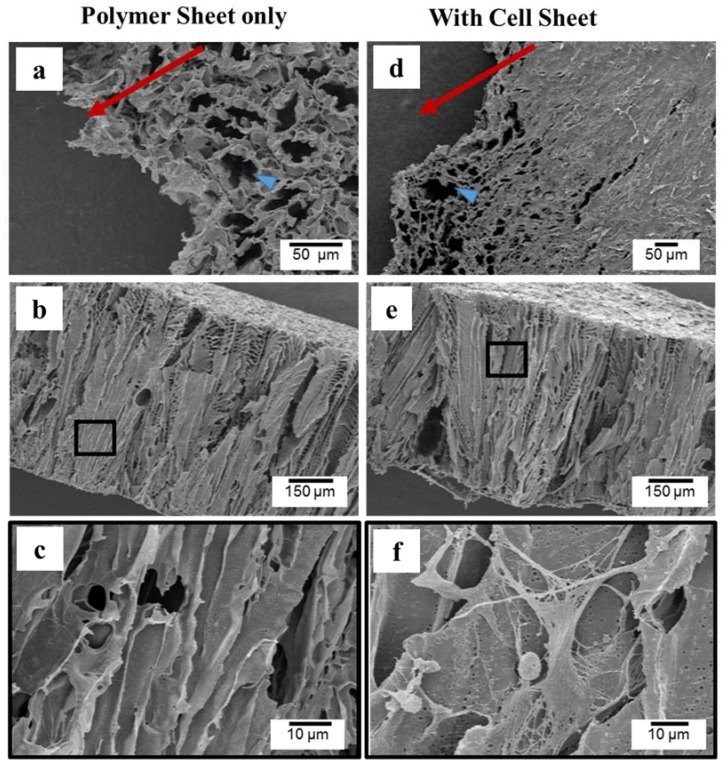
SEM images of deformed structure of 7 days of culture after tensile test. (**a**,**d**) the appearance of pore deformation at break point. Red arrows indicated direction of force. (**b**,**c**,**e**,**f**) fracture appearance in cross section.

SEM images of typical microstructural deformation at the break point are shown in Figure 6. Both of the PLCL sheet only and the PLCL sheet with MSCs exhibited ductile deformation. However, the deformed region of the PLCL sheet with MSCs is wider than that of PLCL sheet only, as indicated in Figure 6a,d (blue head arrow). The cross sectional images showed tearing failure (Figure 6b,e) with deformed pores (Figure 6c,f). It is surprising that the cells can be found inside the elongated pores which formed a connecting network around strut walls (Figure 6f).

## 3. Discussion

One of the key factors in tissue engineered vascular patch is the capability of cellularized patch scaffold to regenerate new tissue and to integrate with native vascular tissue. To encourage the cellularization of patch graft, the scaffold should promote cell migration and cell growing in the three dimensional condition. Therefore, it is necessary to design scaffold with highly porous and interconnected structure. The solid-liquid phase separation method subsequent with the freeze drying method is a facile technique to construct scaffold with suitable porous structure and has been practiced to prepare tissue engineered patch for clinical trial [19]. In the present study, PLCL sheet fabricated by this method had a porous structure with average pore diameter of 21 ± 4.5 µm (Figure 1a–c) which provides an adequate size to support cell infiltration (Figure 3) and proliferation (Figure 4).

Furthermore, to be able to integrate with native vascular tissue, it is important for the tissue engineered patch to beantithrombogenic and resistant to immune response and highly durable. It has been reported that matured endothelial layer on lumen area of vascular graft could maintain good long-term patency [31,32]. The scaffold is seeded with endothelial cells then cultured *in vitro* to allow the formation of endothelial layer on luminal surface of scaffold prior implantation. Therefore, the current approach is potentially applicable for developing antithrombogenic tissue engineered patch. The technique, however, is mainly limited by two factors. First, cell seeding efficiency is low as not all cells attach on the scaffold; second, the extended time to form a mature endothelial cell layer reduce its cost efficiency. In the present study, we tried to overcome these limitations by employing the cell sheet technology with biodegradable scaffold. Since the confluent MSCs can be harvested as an intact layer of MSCs, the seeding efficiency is higher than the manual seeding using dissociated cells [33]. Moreover, MSCs sheet has been reported to repair myocardial infraction [26] and has capacity to differentiate into endothelial cells [27,34]. In this study, MSCs sheet was layered on the porous PLCL sheet and remained attached and intact at 1 day of culture (Figure 3a,d). This combination technique enables us to prepare the mature cell sheet in parallel with scaffold fabrication, thus may allow construction process more efficiently in which usually take additional 1–4 weeks to create endothelium on the scaffold [35].

Apart from that, the combination of cell sheet and porous scaffold could be an alternative way to overcome the cell sheet limitation. Previous study reported a difficulty to construct a thicker cell sheet because such high cell density environment showed necrosis and apoptosis due to lack of nutrient and oxygen supply [28,29,36]. We hypothesized that when cell sheet is combined with scaffold, the porous structure can provide channels for nutrient delivery and metabolic waste removal, thus enabling cell sheet to survive even in high cell density. In the present study, cells from MSCs sheet could migrate into the PLCL sheet through connected porous and formed a new layer of the MSCs sheet on the opposite side of the PLCL sheet. The cross section image of histological staining (Figure 3c) revealed that the cell sheet could reach approximately 54 µm in the thickness direction, without giving any indication of decreasing cell number during cell culture, as supported by the proliferation assays result (Figure 4).

**Figure 7:**
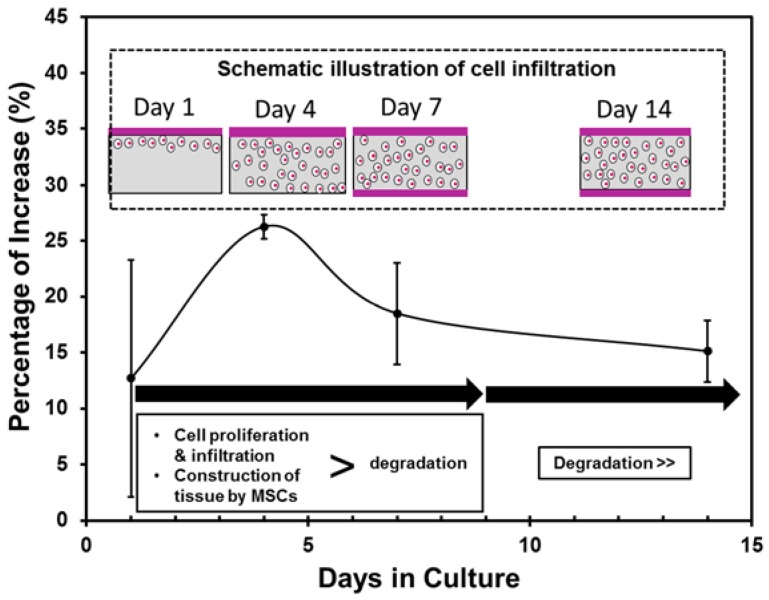
Percentage of increase of tensile strength during 14 days of culture.

This study focused on the influence of MSCs sheet on the mechanical behavior of PLCL sheet. It has been shown that the PLCL sheet with MSCs showed higher elastic modulus at 1 day of culture, however, from 4 to 14 days of culture, the elastic moduli of the PLCL sheet with and without MSCs could maintain similarity (Figure 5). It means that the MSCs sheet is able to maintain the elasticity of the graft. This influence on elasticity was also agreed with the previous study that reported cell sheet of smooth muscle cells improved the elasticity of electrospinned PLCL tubular graft [33]. In contrast, the MSCs sheet gave a variational tensile strength behavior during *in vitro* study which is characterized by the influence of cellular proliferation and infiltration, as summarized in Figure 7. On the initial day of culture, the cells that migrated from the MSCs sheet did not affect strongly the tensile strength change. However, over the next 4 days of culture, cells were actively proliferating in the inner region and penetrated entirely into the pores, thus increase tensile strength around 26%. This kind of improvement is thought to be the effect of increasing cell number which support on the collagen production, leading to increasing tensile strength [37,38]. At 7 days of culture, a unique, layered, sandwich-like structure was observed, with cell sheets on both the surface and the bottom of the polymer scaffold in the center. This layered construct had a tensile strength significantly greater than the PLCL sheet only although percentage of increase decreased. At 14 days of culture, although cells continued to proliferate and formed a more mature layered construct, the degradation of the PLCL sheet affected the decrease of tensile strength greater than the strengthening effect by cellular infiltration. This degradation was supported by the weight loss data of the PLCL sheet during incubation (Figure 5f). In 2 weeks, the PLCL sheet had lost approximately 4% of its weight. This result suggested that hydrolytic degradation might have caused a significant structure erosion and strut thinning, thus affected greatly the decreasing tensile strength.

The increasing material density due to cell penetration into the pores is considered to have an effect on the increase of strain energy density greatly particularly at day 4 and 7 of culture (Figure 5e). The deformation mechanism of both PLCL sheet with and without MSCs were characterized by ductile tearing failure of strut under the tensile test. Both of them showed elongated pores at the critical stress, however, the deformation region of the PLCL sheet with MSCs is wider than the PLCL, having an influence on the increase of strain energy density increasing (Figure 6).

Finally, the described results demonstrates two important points. First, combining cell sheet with porous scaffold could be one of promising ways to preserve the critical properties of cells as well as provide better environment for tissue growth. Second, the results suggested that cell sheet could enhance the tensile strength and strain energy density of the PLCL sheet thus may prolong biomechanical stability when implanted *in vivo*. As the next step, layering endothelial cells sheet on the outer layer of porous tubular scaffold is currently under development to observe whether cell sheet can migrate on the lumen of tubular graft, resulting an engineered vascular with endothelium on lumen area.

## 4. Conclusions

In this study we have developed new technique to construct a unique tissue engineered patch by layering MSCs sheet on porous PLCL sheet. The conclusions were obtained as follows:

1. MSCs sheet was able to survive (even in a thicker density) and actively proliferate on the porous PLCL sheet, suggesting that the porous structure is important in transporting nutrient and oxygen supply while removing metabolic waste.
2. Variations in the tensile strength of the layered constructs were affected by two factors, the cell proliferation and infiltration and the degradation of the PLCL sheet. Once the cell sheet proliferated entirely into the pores of the PLCL sheet and formed a new layer on the opposite surface, the layered construct exhibited significantly higher tensile strength than that without MSCs. However, after 14 days of culture the effect of the degradation of the PLCL sheet overtook that of cell proliferation, resulting the decrease of the tensile strength.

## 5. Materials and Methods

### 5.1. Fabrication of PLCL Sheet

Porous tubular scaffolds composed of (PLCL) at 75:25 ratio (Gunze Ltd., Kyoto, Japan) were fabricated by the solid liquid phase separation method followed by the freeze-drying method under vacuum [19]. The polymer granules were dissolved in 1.4-dioxane solvent with final concentration of 6% (w/v). A Teflon tube of 10 mm in diameter taken from −80 °C was vertically dipped into the polymer solution and pulled out at a constant rate (100 mm/min). This sample was frozen again at −80 °C for at least 1 h before being freeze-dried at −50 °C for 24 h. Finally, the graft was pulled out from the Teflon tubes and cut into sheets as shown in Figure 1a. To evaluate the degradation process, the PLCL sheet was incubated in a phosphate buffer saline (PBS) solution at 37 °C/5% CO_2_ for 1, 4, 7 and 14 days. The percentage of weight loss of PLCL sheet was calculated by comparing the weight before incubation with the weight after incubation.

### 5.2. Fabrication of Layered Construct

Human MSCs (UE6E7TE, Riken BRC, Tsukuba, Japan) of passage 4–5 were cultured on a 24-multi well temperature-responsive dish (CellSeed Inc., Tokyo, Japan) at a density of 5 × 10^4^ cells/well. The cells were cultured in cell growth medium containing α-MEM (Wako Chem.,Tokyo, Japan), 10% FBS, and 1% penicillin streptomycin in a humidified atmosphere with 5% CO_2_ at 37 °C for 4 days. The cell sheets were obtained by detaching the confluent cells from the dishes at room temperature for 20 min. Each cell sheet was transferred on to a PLCL sheet (10 mm × 10 mm) which had been sterilized by ethanol 70% and ultraviolet. After removing media, the layered construct was incubated for 1 h to promote adhesion between the cell sheet and the PLCL sheet. Finally, the layered construct was incubated in 2 mL of cell growth medium and cultured for 2 weeks.

### 5.3. SEM Imaging

The microstructure of each sample was observed using a field-emission scanning electron microscope (FE-SEM) (Hitachi Ltd., Tokyo, Japan, S-4100). For the layered construct, the sample was washed with phosphate buffer saline, dehydrated in ascending concentration of ethanol (40%, 50%, 70% and 100%) and soaked in t-butyl alcohols. Then the sample was freeze-dried in a freeze drying machine (ES-2040, Hitachi, Ltd., Tokyo, Japan) and sputter-coated with Pt–Pd using an anion sputter coater (E-1040, Hitachi, Ltd., Tokyo, Japan). Both the cross section and surface area were observed.

### 5.4. Cell Proliferation

The cell viability of each layered construct was evaluated by using a Cell Counting Kit (Dojindo Laboratories, Kumamoto, Japan). The layered construct was soaked in PBS and the cell counting kit, and incubated in a CO_2_ incubator for 2 h. The absorbance was measured by a spectrophotometric plate reader (2040 ARVO™ X2, Perkin Elmer Co., Yokohama, Japan) at a wavelength of 450 nm.

### 5.5. H&E Staining

After incubating for 1, 4, 7, and 14 days, the layered construct was recovered from the dish, washed with PBS, and dehydrated with ascending ethanol solution (40%, 50%, and 70%). The layered construct was embedded in paraffin, sectioned and stained with Hematoxylin and Eosin (H&E). The image was observed using a microscope digital camera (Olympus DP12, Olympus Optical Co., Ltd., Tokyo, Japan).

### 5.6. Mechanical Testing

Tensile mechanical tests were performed after 1, 4, 7, and 14 days of cell cultures by using a Shimadzu Compact Tabletop Testing Machine with a 10 N load cell and a crosshead speed of 1 mm/min. The region under measurement was rectangular in shape (10 mm in length, 10 mm in width, 0.5 mm in thickness). Based on the load-displacement relation that was monitored during the test, the stress (σ) and strain (ε) were evaluated using the following formulas.

(1) $$\sigma =\frac{F}{A}$$

(2) $$\varepsilon =\frac{∆L}{L}$$

where *F* is the force under tensile test, *A* is the area of cross sectional sample, L is the length of sample under measurement and ∆L is the displacement after loading at each time. The elastic modulus was calculated by identifying the linear region in the resulting stress-strain curve.

### 5.7. Statistical Analysis

All data were expressed as mean ± SD. Statistical analysis were performed by Fischer’s LCD comparison test in which *p* values of less than 0.05 were considered statistically significant.

## References

[1] Sun R., Liu M., Lu L., Zheng Y., Zhang P. (2015). Congenital heart disease: Causes, diagnosis, symptoms, and treatments. Cell Biochem. Biophys..

[2] Avolio E., Caputo M., Madeddu P. (2015). Stem cell therapy and tissue engineering for correction of congenital heart disease. Front. Cell Dev. Biol..

[3] Ozawa T., Mickle D.A.G., Weisel R.D., Koyama N., Ozawa S., Li R.-K. (2002). Optimal biomaterial for creation of autologous cardiac grafts. Circulation.

[4] Ozawa T., Mickle D.A.G., Weisel R.D., Koyama N., Wong H., Ozawa S., Li R.-K. (2002). Histologic changes of nonbiodegradable and biodegradable biomaterials used to repair right ventricular heart defects in rats. J. Thorac. Cardiovasc. Surg..

[5] Mirensky T.L., Breuer C.K. (2008). The development of tissue-engineered grafts for reconstructive cardiothoracic surgical applications. Pediatr. Res..

[6] Lee W.K., Park K.D., Kim Y.H., Suh H., Park J.C., Lee J.E., Sun K., Baek M.J., Kim H.-M., Kim S.H. (2001). Improved calcification resistance and biocompatibility of tissue patch grafted with sulfonated peo or heparin after glutaraldehyde fixation. J. Biomed. Mater. Res..

[7] Said S.M., Burkhart H.M. (2014). When repair is not feasible: Prosthesis selection in children and adults with congenital heart disease. Semin. Thorac. Cardiovasc. Surg. Pediatr. Card. Surg. Annu..

[8] Schmidt C.E., Baier J.M. (2000). Acellular vascular tissues: Natural biomaterials for tissue repair and tissue engineering. Biomaterials.

[9] Okwulehie V., Dharmapuram A.K., Swain S.K., Ramdoss N., Sundararaghavan S., Kona S.M. (2006). Experience with autologous pericardial patch closure of ventricular septal defect. Indian J. Thorac. Cardiovasc. Surg..

[10] Pok S., Jacot J.G. (2011). Biomaterials advances in patches for congenital heart defect repair. J. Cardiovasc. Transl. Res..

[11] Schmidt D., Mol A., Neuenschwander S., Breymann C., Gössi M., Zund G., Turina M., Hoerstrup S.P. (2005). Living patches engineered from human umbilical cord derived fibroblasts and endothelial progenitor cells. Eur. J. Cardiothorac. Surg..

[12] Hoerstrup S.P., Kadner A., Breymann C., Maurus C.F., Guenter C.I., Sodian R., Visjager J.F., Zund G., Turina M.I. (2002). Living, autologous pulmonary artery conduits tissue engineered from human umbilical cord cells. Ann. Thorac. Surg..

[13] Cho S.-W., Jeon O., Lim J.E., Gwak S.-J., Kim S.-S., Choi C.Y., Kim D.-I., Kim B.-S. (2006). Preliminary experience with tissue engineering of a venous vascular patch by using bone marrow–derived cells and a hybrid biodegradable polymer scaffold. J. Vasc. Surg..

[14] Yeong W.Y., Sudarmadji N., Yu H.Y., Chua C.K., Leong K.F., Venkatraman S.S., Boey Y.C.F., Tan L.P. (2010). Porous polycaprolactone scaffold for cardiac tissue engineering fabricated by selective laser sintering. Acta Biomater..

[15] Serrano M.C., Pagani R., Ameer G.A., Vallet-Regi M., Portoles M.T. (2008). Endothelial cells derived from circulating progenitors as an effective source to functional endothelialization of naoh-treated poly(epsilon-caprolactone) films. J. Biomed. Mater. Res. A.

[16] Ye L., Cao J., Chen L., Geng X., Zhang A.Y., Guo L.R., Gu Y.Q., Feng Z.G. (2015). The fabrication of double layer tubular vascular tissue engineering scaffold *via* coaxial electrospinning and its 3d cell coculture. J. Biomed. Mater. Res. A.

[17] Garkhal K., Verma S., Tikoo K., Kumar N. (2007). Surface modified poly(l-lactide-co-epsilon-caprolactone) microspheres as scaffold for tissue engineering. J. Biomed. Mater. Res. A.

[18] Lakshmanan R., Krishnan U.M., Sethuraman S. (2012). Living cardiac patch: The elixir for cardiac regeneration. Expert Opin. Biol. Ther..

[19] Shin’oka T., Matsumura G., Hibino N., Naito Y., Watanabe M., Konuma T., Sakamoto T., Nagatsu M., Kurosawa H. (2005). Midterm clinical result of tissue-engineered vascular autografts seeded with autologous bone marrow cells. J. Thorac. Cardiovasc. Surg..

[20] Hibino N., McGillicuddy E., Matsumura G., Ichihara Y., Naito Y., Breuer C., Shinoka T. (2010). Late-term results of tissue-engineered vascular grafts in humans. J. Thorac. Cardiovasc. Surg..

[21] Mescher A.L., Mescher A.L., Junqueira L.C.U.A. (2016). Junqueira’s Basic Histology: Text and Atlas.

[22] Cho S.-W., Park H.J., Ryu J.H., Kim S.H., Kim Y.H., Choi C.Y., Lee M.-J., Kim J.-S., Jang I.-S., Kim D.-I. (2005). Vascular patches tissue-engineered with autologous bone marrow-derived cells and decellularized tissue matrices. Biomaterials.

[23] Matsuda N., Shimizu T., Yamato M., Okano T. (2007). Tissue engineering based on cell sheet technology. Adv. Mater..

[24] Okano T., Yamada N., Sakai H., Sakurai Y. (1993). A novel recovery system for cultured cells using plasma-treated polystyrene dishes grafted with poly(*n*-isopropylacrylamide). J. Biomed. Mater. Res..

[25] Kushida A., Yamato M., Konno C., Kikuchi A., Sakurai Y., Okano T. (1999). Decrease in culture temperature releases monolayer endothelial cell sheets together with deposited fibronectin matrix from temperature-responsive culture surfaces. J. Biomed. Mater. Res..

[26] Miyahara Y., Nagaya N., Kataoka M., Yanagawa B., Tanaka K., Hao H., Ishino K., Ishida H., Shimizu T., Kangawa K. (2006). Monolayered mesenchymal stem cells repair scarred myocardium after myocardail infarction. Nature.

[27] Oswald J., Boxberger S., Jørgensen B., Feldmann S., Ehninger G., Bornhäuser M., Werner C. (2004). Mesenchymal stem cells can be differentiated into endothelial cells *in vitro*. Stem Cells.

[28] Shimizu T., Sekine H., Yang J., Isoi Y., Yamato M., Kikuchi A., Kobayashi E., Okano T. (2006). Polysurgery of cell sheet grafts overcomes diffusion limits to produce thick, vascularized myocardial tissues. FASEB J..

[29] Sekine W., Haraguchi Y., Shimizu T., Umezawa A., Okano T. (2011). Thickness limitation and cell viability of multilayered cell sheets and overcoming the diffusion limit by a porous-membrane culture insert. J. Biochip Tissue Chip.

[30] Suzuki R., Hattori F., Itabashi Y., Yoshioka M., Yuasa S., Manabe-Kawaguchi H., Murata M., Makino S., Kokaji K., Yozu R. (2009). Omentopexy enhances graft function in myocardial cell sheet transplantation. Biochem. Biophys. Res. Commun..

[31] Ratcliffe A. (2000). Tissue engineering of vascular grafts. Matrix Biol..

[32] Cleary M.A., Geiger E., Grady C., Best C., Naito Y., Breuer C. (2012). Vascular tissue engineering: The next generation. Trends Mol. Med..

[33] Ahn H., Ju Y.M., Takahashi H., Williams D.F., Yoo J.J., Lee S.J., Okano T., Atala A. (2015). Engineered small diameter vascular grafts by combining cell sheet engineering and electrospinning technology. Acta Biomater..

[34] Li Q., Wang Z. (2013). Influence of mesenchymal stem cells with endothelial progenitor cells in co-culture on osteogenesis and angiogenesis: An *in vitro* study. Arch. Med. Res..

[35] Tondreau M.Y., Laterreur V.R., Gauvin R., ValliAres K., Bourget J.-M., Lacroix D., Tremblay C., Germain L., Ruel J., Auger F.O.A. (2015). Mechanical properties of endothelialized fibroblast-derived vascular scaffolds stimulated in a bioreactor. Acta Biomater..

[36] Sekine W., Haraguchi Y., Shimizu T., Yamato M., Umezawa A., Okano T. (2013). Chondrocyte differentiation of human endometrial gland-derived mscs in layered cell sheets. Sci. World J..

[37] Mun C.H., Jung Y., Kim S.-H., Kim H.C., Kim S.H. (2013). Effects of pulsatile bioreactor culture on vascular smooth muscle cells seeded on electrospun poly(lactide-co-ε-caprolactone) scaffold. Artif. Organs.

[38] Sang B.S., Jung Y., Kwon I.K., Hay M.C., Kim S.H. (2012). Fibroblast culture on poly(l-lactide-co-ε-caprolactone) an electrospun nanofiber sheet. Macromol. Res..

